# P–C-Activated Bimetallic Rhodium Xantphos Complexes: Formation and Catalytic Dehydrocoupling of Amine–Boranes**

**DOI:** 10.1002/anie.201504073

**Published:** 2015-07-03

**Authors:** Heather C Johnson, Andrew S Weller

**Affiliations:** Department of Chemistry, University of Oxford Mansfield Road, Oxford, OX1 3TA (UK) E-mail: andrew.weller@chem.ox.ac.uk

**Keywords:** amine–boranes, homogeneous catalysis, P ligands, rhodium, X-ray diffraction

## Abstract

{Rh(xantphos)}-based phosphido dimers form by P–C activation of xantphos (4,5-bis(diphenylphosphino)-9,9-dimethylxanthene) in the presence of amine–boranes. These dimers are active dehydrocoupling catalysts, forming polymeric [H_2_BNMeH]_*n*_ from H_3_B⋅NMeH_2_ and dimeric [H_2_BNMe_2_]_2_ from H_3_B⋅NMe_2_H at low catalyst loadings (0.1 mol %). Mechanistic investigations support a dimeric active species, suggesting that bimetallic catalysis may be possible in amine–borane dehydropolymerization.

The catalytic dehydrocoupling of amine–boranes[1] has received much attention with regard to hydrogen storage applications (e.g. H_3_B⋅NH_3_)[2] or as a means to form BN-based materials, such as polyaminoboranes [H_2_BNRH]_*n*_ (R=H, Me, *n*Bu), through catalytic dehydropolymerization.[3] We have recently reported the complex [Rh(κ^2^-_P,P_-xantphos){η^2^-H_2_B(CH_2_CH_2_*t*Bu)⋅NMe_3_}][BAr^F^_4_] **1** (Ar^F^=3,5-(CF_3_)_2_C_6_H_3_, xantphos=4,5-bis(diphenylphosphino)-9,9-dimethylxanthene), which acts as a source of the {Rh(xantphos)}^+^ fragment.[4] Complex **1** is an active catalyst for the dehydropolymerization of H_3_B⋅NMeH_2_ to form [H_2_BNMeH]_*n*_ of moderate molecular weight (*M*_n_=22 700 g mol^−1^, PDI=2.1; PDI=polydispersity index), as well as for the dehydrocoupling of H_3_B⋅NMe_2_H to form [H_2_BNMe_2_]_2_ (Scheme 1 A).[4] Kinetic data indicate an induction period involving rate limiting N–H activation and that saturation kinetics operate during productive catalysis, suggesting an active catalyst that has an amidoborane motif which binds a subsequent equivalent of amine–borane. However, the precise structure of this active species remains unclear. Complex **1** also promotes the stoichiometric B–B homocoupling of H_3_B⋅NMe_3_ to form [Rh(κ^2^-_P,P_-xantphos)(H_4_B_2_⋅2NMe_3_)][BAr^F^_4_] **2** alongside the dihydride complex [Rh(κ^3^-_P,O,P_-xantphos)(H)_2_(η^1^-H_3_B⋅NMe_3_)][BAr^F^_4_] **3** (Scheme 1 B)[5] and also promotes the hydroboration of alkenes using H_3_B⋅NMe_3_.[6]

**Scheme 1:**
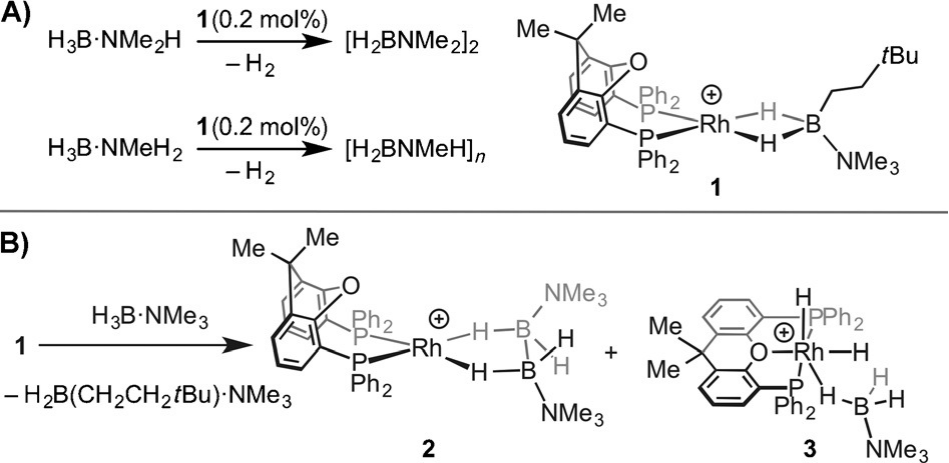
A) Catalytic dehydrocoupling of H_3_B⋅NMe_2_H and H_3_B⋅NMeH_2_ with 1. B) Homocoupling of H_3_B⋅NMe_3_ using 1. [BAr^F^_4_]^−^ ions are not shown.

We now report that at the end of amine–borane dehydrocoupling using catalyst **1**, Rh_2_ dimeric complexes are isolated in which a xantphos ligand has undergone P–C activation.[7] Surprisingly this dimeric motif also acts a very active precatalyst, suggesting the possibility for cooperative bimetallic reactivity[8] in the dehydropolymerization of amine–boranes.[9]

Addition of **1** (5 mol %) to H_3_B⋅NMe_2_H in a 1,2-C_6_H_4_F_2_ solution (sealed NMR tube) resulted in the rapid formation of a Rh^III^ dihydride sigma amine–borane complex, an induction period before the onset of catalysis, and the observation (65 % consumption, 3 h) of a resting state tentatively assigned to an amidoborane complex.[4] Investigation of the reaction mixture at a late stage of the catalytic process (>90 % consumption, 4.5 h) revealed a new complex that became the major organometallic product after 12 h (Scheme 2). Isolation as an orange crystalline material in 40 % yield (based on Rh), and analysis by single-crystal X-ray diffraction, NMR spectroscopy, and ESI-MS show this to be a xantphos-derived P–C-activated phosphido-bridged Rh_2_ dimer with a coordinated *N*,*N*-dimethylaminodiboranate: [Rh_2_(κ^2^-_P,P_-xantphos′)_2_(η^2^,η^2^-H_3_BNMe_2_BH_3_)][BAr^F^_4_] **4** (Figure 1).

**Figure 1:**
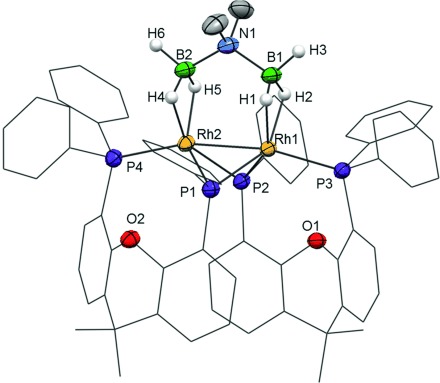
Solid-state structure of the cationic component of 4. Displacement ellipsoids are set at 50 % probability. H atoms bound to C centers are omitted for clarity, and the carbon atoms in the xantphos′ ligands are depicted as a wireframe. Selected bond lengths [Å] and angles [°]: Rh1–Rh2 2.5928(4), Rh1–P1 2.2455(12), Rh1–P2 2.2500(11), Rh1–P3 2.3427(11), Rh2–P1 2.2461(11), Rh2–P2 2.2663(11), Rh2–P4 2.3325(11), Rh1–B1 2.234(5), Rh2–B2 2.229(5), Rh1–O1 3.393(4), Rh2–O2 3.412(4), B1–N1 1.600(7), N1–B2 1.583(7); P1-Rh2-P4 114.64(4), P2-Rh1-P3 110.89(4), B1-N1-B2 117.5(4).

**Scheme 2:**
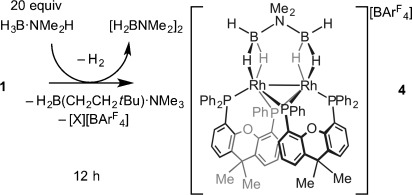
The formation of complex 4 at 5 mol % catalyst loadings. The identity of [X][BAr^F^_4_] was not determined.

These data reveal that the xantphos ligands have undergone a P–C activation, forming two bridging phosphido groups (4-phenylphosphido-5-diphenylphosphino-9,9-dimethylxanthene, xantphos′) which span the Rh–Rh vector (Figure 1). Each xantphos′ ligand is bound in a κ^2^-P,P configuration and the central Rh_2_P_2_ unit adopts a butterfly geometry.[10] A [H_3_BNMe_2_BH_3_]^−^ ion[11, 12] bridges the two metal centers. The B–H hydrogen atoms were located, and each BH_3_ group is bound η^2^ to the metal center (Rh–B 2.234(5), 2.229(5) Å). A Rh–Rh single bond is proposed,[13] and although the distance of 2.5928(4) Å might thus be considered short,[14] the Rh_2_P_2_ unit appears fairly flexible to the requirements of the bridging ligands as the Rh–Rh distances in complexes **5** and **7** (see below) are longer. The NMR spectroscopic data (CD_2_Cl_2_) are fully consistent with the solid-state structure. Two environments are detected in the ^31^P{^1^H} NMR spectrum with resonance signals at *δ*=108.5 (virtual tt) and *δ*=13.3 ppm (virtual ddt) assigned to the phosphido and phosphino groups, respectively, on the basis of chemical shifts and coupling constants.[15, 16] The ^11^B NMR spectrum shows a broad resonance at *δ*=16.6 ppm corresponding to the BH_3_ groups, shifted circa 29 ppm downfield compared to Na[H_3_B⋅NMe_2_⋅BH_3_] (*δ*=−12.5 ppm, q, THF),[11] suggesting a significant interaction with the metal.[17] Signals attributable to the bridging Rh–H–B hydrides in **4** are detected at *δ*=−2.68 and *δ*=−3.52 ppm in the ^1^H NMR spectrum, whereas signals for the terminal B–H groups are detected at *δ*=4.14 ppm. These signals sharpen in the ^1^H{^11^B} NMR spectrum, and the observation of three different environments in a 1:1:1 ratio shows that the BH_3_ unit is static on the NMR timescale. Complex **4** is best considered as a 26-electron {Rh(κ^2^-_P,P_-xantphos′)_2_}^2+^ fragment coordinated with [H_3_BNMe_2_BH_3_]^−^. Bimetallic complexes with bridging amine- or phosphine–borane-derived ligands are rare.[9b, 18]

The P–C-activated {Rh(xantphos′)}_2_^2+^ motif observed in **4** can also be formed by heating a 1:1 mixture of complexes **2** and **3** (Scheme 1, generated in situ[5]) at 40 °C for 6 days. This reaction results in the formation, in quantitative yield (determined by NMR spectroscopy), of [Rh_2_(κ^3^-_P,O,P_-xantphos′)_2_(η^1^-H_3_B⋅NMe_3_)_2_][BAr^F^_4_]_2_ **5** (Scheme 3). Although similar to **4**, the solid-state structure (Figure 2) shows that each H_3_B⋅NMe_3_ binds in a η^1^ configuration with the metal center and the xantphos′ adopts a κ^3^-P,O,P bonding motif. There is a crystallographically imposed twofold axis of symmetry in the cation. The Rh–Rh distance of 2.7965(5) Å is significantly longer than in **4** (2.5928(4) Å), reflecting the difference in monodentate and bridging ligands respectively, whereas the Rh–B distance of 2.722(4) Å shows η^1^ amine–borane binding. The solution NMR data for **5** are consistent with the solid-state structure.[16] In the ^31^P{^1^H} NMR spectrum two environments are detected, *δ*=135.1 (vitual tt) and *δ*=19.2 ppm (virtual ddt), whereas in the ^1^H NMR spectrum signals for the H_3_B groups are detected as a broad signal at *δ*=−0.39 ppm (relative integral 6 H). This suggests rapid exchange between the bridging and terminal B–H groups, and cooling to 200 K did not result in splitting of this signal. The ^11^B NMR spectrum displays a broad signal at *δ*=−7.8 ppm, barely shifted from that of free H_3_B⋅NMe_3_. All these data are consistent with η^1^-coordination of the amine–borane being retained in solution.[17, 19] Complex **4** can also be formed by reaction of **5** with Na[H_3_BNMe_2_BH_3_], revealing that the xantphos′ ligand can adopt a flexible coordination mode[20] in response to the requirements of the amine–borane ligands.

**Figure 2:**
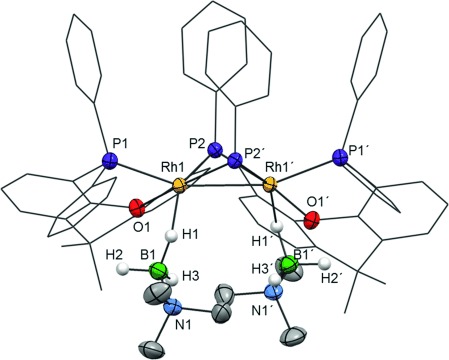
Solid-state structure of the cationic component of 5. Displacement ellipsoids are set at 50 % probability. H atoms bound to C centers are omitted, and the carbon atoms in the chelating ligand backbone are depicted as a wireframe. Selected bond lengths [Å] and angles [°]: Rh1–Rh1′ 2.7965(5), Rh1–P2′ 2.1940(9), Rh1–P2 2.2192(8), Rh1–P1 2.3344(9), Rh1–O1 2.288(2), Rh1–B1 2.722(4), N1–B1 1.594(5); P2-Rh1-P1 119.72(3), Rh1-P2-Rh1′ 78.64(3), Rh1′-Rh1-P2′ 51.08(2), Rh1′-Rh1-P2 50.28(2).

**Scheme 3:**
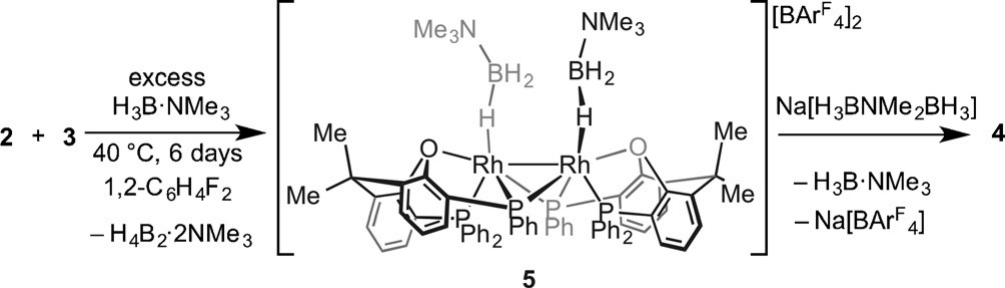
Formation of complex 5 and subsequent reaction to form complex 4.

The σ-bound H_3_B⋅NMe_3_ ligands in **5** can also be easily displaced by addition of MeCN or Ph_2_PCH_2_CH_2_PPh_2_ (dppe) to form [Rh_2_(κ^3^-_P,O,P_-xantphos′)_2_(L)_2_][BAr^F^_4_]_2_ **6** (L=MeCN) or **7** (L_2_=dppe), respectively (Scheme 4). In both cases, the Rh_2_-based dimeric unit remains intact, as shown by NMR spectroscopy. The solid-state structure of **7** confirms the {Rh(xantphos′)}_2_ motif, showing a κ^3^-P,O,P geometry and a bridging dppe ligand.[16] The Rh–Rh distance in **7** is 2.8362(5) Å, longer than in both **4** and **5**. Addition of H_3_B⋅NMe_2_H (4 equiv) to **5** results in dehydrocoupling to form [H_2_BNMe_2_]_2_ and a 1:1 mixture of **4**:**5**.

**Scheme 4:**
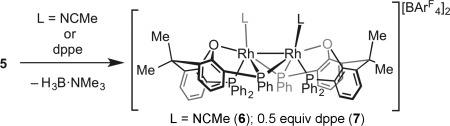
Formation of complexes 6 and 7.

Although not fully resolved, there are clues to the mechanism of formation of these dimers. During the formation of **5**, no intermediates were detected by NMR spectroscopy, whereas benzene and biphenyl were detected in a circa 3:1 ratio by GC–MS in the final reaction mixture, consistent with P-aryl bond cleavage and subsequent elimination. The requirement for both Rh^I^ and Rh^III^ fragments is demonstrated by the fact that heating Rh^I^-based complex **2** to 40 °C with excess H_3_B⋅NMe_3_ for one week (1,2-C_6_H_4_F_2_) did not produce **5**, but addition of **3** to excess H_3_B⋅NMe_3_ formed **5** quantitatively (6 days, 40 °C), during which signals for the formation of small amounts of **2** were detected by NMR spectroscopy. Under an atmosphere of H_2_ (4 atm), which would favor Rh^III^ dihydride species,[4] the conversion is slower, roughly 30 % in 6 days. Combination of [Rh(κ^3^-_P,O,P_-xantphos)(H)_2_(NCMe)][BAr^F^_4_][21] and [Rh(κ^3^-_P,O,P_-xantphos)(PCy_3_)][BAr^F^_4_][6] at 40 °C for 5 days resulted in essentially no change, which suggests that rather labile ligands and/or amine–borane are required for dimer formation. We speculate that low-coordinate Rh^I^ {Rh(xantphos)}^+^ and Rh^III^ {Rh(xantphos)(H)_2_}^+^ fragments formed in situ combine to form a complex with bridging hydrides, such as, for example, [Rh_2_(xantphos)_2_(μ-H)_2_(H_3_B⋅NMe_3_)_*n*_][BAr^F^_4_]_2_. From this, P–C activation of xantphos occurs with subsequent elimination of benzene or biphenyl/H_2_. Although there are many reports of the P–C activation of phosphines to form phosphido bridges,[7, 22] this is, to our knowledge, the first instance of such a process occurring with the xantphos ligand. P–C activation of xantphos has been reported from [Pd(κ^3^-_P,O,P_-xantphos)(*closo*-SnB_11_H_11_)] to form a direct P–B bond.[23]

The formation of phosphido bridges during homogeneous catalysis is generally considered a catalyst deactivation route.[7, 25] However, examples of such species acting as precatalysts in a variety of transformations are known,[26] and there is evidence suggesting that in this system dimers do play a role. The dehydrocoupling of H_3_B⋅NMe_2_H catalyzed by **5** was investigated using conditions which were the same as those described for monomer precatalyst **1** (0.2 mol %, open system).[4] Addition of **5** (0.1 mol %; i.e. 0.2 mol % [Rh]) to H_3_B⋅NMe_2_H formed [H_2_BNMe_2_]_2_ with H_2_B=NMe_2_ as the major boron-containing intermediate detected (Figure 3 A). There is an induction period of circa 300 s prior to productive catalysis (turnover frequency (TOF) 2300 h^−1^, 1150 h^−1^ relative to [Rh]; compared with circa 1000 h^−1^ using **1**). Turnover continued after addition of excess Hg to the reaction mixture, suggesting homogeneous catalysis. Upon varying the initial [H_3_B⋅NMe_2_H] across the range 0.288–0.018 m, system behavior broadly suggestive of saturation kinetics was measured (post-induction period): at [H_3_B⋅NMe_2_H] above circa 0.1 m, pseudo-zero-order decay of [H_3_B⋅NMe_2_H] was measured, while approximate pseudo-first-order consumption of H_3_B⋅NMe_2_H was found at lower concentrations (Figure 3 B). Similar saturation kinetics were previously shown with **1**.[4] During the early stages of catalysis, post-induction, the measured rate shows a first order dependence on [**5**], rather than half order that would suggest a rapid dimer–monomer equilibrium in which the dimer lies off-cycle.[27]

**Figure 3:**
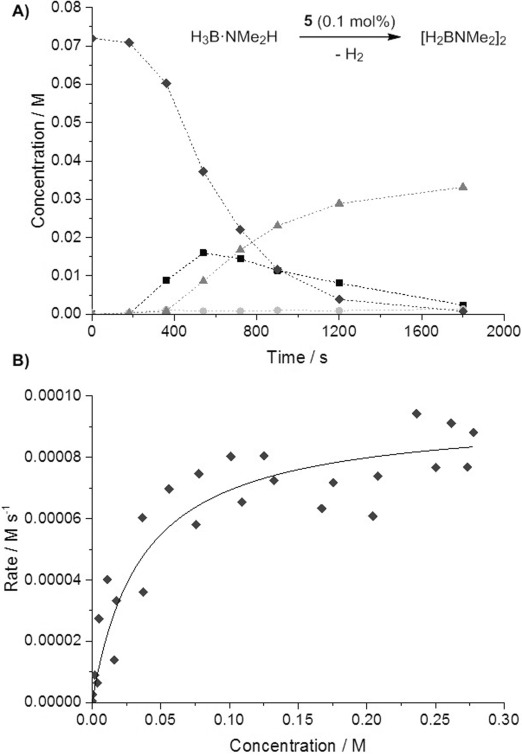
A) Concentration versus time plots (as measured by ^11^B NMR spectroscopy) for species H_3_B⋅NMe_2_H (♦, black), H_2_B=NMe_2_ (▪, black), HB(NMe_2_)_2_ (•, light gray), and [H_2_BNMe_2_]_2_ (▴, dark gray). Conditions: [H_3_B⋅NMe_2_H]_0_=0.072 m, [5]=7.2×10^−5^ m, 1,2-C_6_H_4_F_2_ was used as solvent, open conditions, 298 K. B) Plot of rate versus [H_3_B⋅NMe_2_H] for the dehydrocoupling of [H_3_B⋅NMe_2_H] by 5, showing the post-induction period. Separate experiments spanning the range 0.288–0.018 m were carried out. The line of best fit is to guide the eye only.[24]

Kinetic isotope effects of 1.1±0.2 and 2.0±0.3 were measured by using D_3_B⋅NMe_2_H and H_3_B⋅NMe_2_D respectively (zero-order regions), suggesting that N–H activation may be involved in, or in an equilibrium prior to, the turnover-limiting step. Moreover, the induction period approximately doubled from about 300 s with H_3_B⋅NMe_2_H and D_3_B⋅NMe_2_H to circa 600 s with H_3_B⋅NMe_2_D, indicating that N–H activation is also involved in the rate-limiting process during the formation of the catalytically active species. Consistent with labelling experiments, B–H activation in **5** is fast and reversible as addition of D_2_ to **5** results in incorporation of D into the BH_3_ groups within the time of mixing, possibly via a σ-CAM-type process (σ-CAM=σ-complex-assisted metathesis).[28] These observations combined suggest that the active catalytic species contains an N–H-activated amine–borane, possibly an amidoborane complex, that quasi-reversibly coordinates a second equivalent of H_3_B⋅NMe_2_H. The dehydrocoupling of H_3_B⋅NMe_2_H (0.072 m, [**5**]=7.2×10^−5^ m) in a sealed system, which enables build-up of H_2_, shows a TOF of circa 450 h^−1^ (relative to [Rh]), slower than in the open system, indicating inhibition by H_2_. The decay of [H_3_B⋅NMe_2_H] follows a first-order profile (post-induction period), again very similar to that seen with **1** under the same conditions.[4] Complex **4** is also active in catalysis, but shows a longer induction period and a slower overall rate.[16] Complex **5** (0.1 mol %) also catalyzes the dehydropolymerization of H_3_B⋅NMeH_2_ to form [H_2_BNMeH]_*n*_ (*M*_n_=28 700 g mol^−1^, PDI=1.7, Scheme 5). This is a similar molecular weight to the [H_2_BNMeH]_n_ produced under analogous conditions with **1** (*M*_n_=22 700 g mol^−1^, PDI=2.1).[4]

**Scheme 5:**
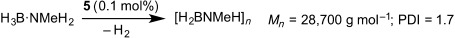
Dehydropolymerization of H_3_B⋅NMeH_2_ using 5 as a catalyst.

Overall, these studies suggest that the mechanism of the dehydrocoupling of amine–boranes by **5** and **1** at low catalyst loadings (0.2 mol % Rh) are likely closely related: both **1** and **5** show induction periods as well as very similar kinetic profiles and isotope effects. We postulate that the active species, whether a monomer or dimer, is accessed through N–H activation. Complex **4** that was isolated at the end of catalysis could form from the addition of BH_3_ to a [Rh]_2_-NMe_2_BH_3_ unit. As shown in Scheme 6, we propose that the active species forms by one of two pathways: a) P–C activation in a Rh^III^ complex related to **3** (which would result from addition of H_3_B⋅NMe_2_H to **1**[4]) that is very fast under conditions of a high relative amine–borane concentration and forms a dimeric species related to **5** that then undergoes a slower N–H activation to form a dimeric active species; or b) the monomeric species are the active catalysts, which could be formed when starting from **5** by opening up of the phosphido bridges, perhaps by protonation by the amine–borane. To explore this latter possibility, complex **5** was reacted with HCl (Et_2_O solution) and MeI. In both cases intractable mixtures resulted. Unfortunately ESI-MS, or crossover experiments using different xantphos-containing precursors, have not been definitive in discounting either the dimer or the monomer as the active species. Catalysis in a sealed NMR tube with 20 equivalents of H_3_B⋅NMe_2_H (5 mol % [Rh]) was employed to probe likely resting states. During catalysis, ^1^H and ^31^P{^1^H} NMR spectra showed broad unresolved signals, suggestive of several species, while after 12 h, complex **4** was again the major organometallic product formed. Under these conditions of higher catalyst loading, dimer **5** is strikingly faster than monomer **1** (TOF 240 h^−1^ and 4 h^−1^,[4] respectively), whereas the dehydrocoupling at much higher relative ratios of amine–borane operate at similar rates (see above; 500 equivalents, 0.2 mol % [Rh], TOF 1150 h^−1^ and 1000 h^−1^, respectively). This difference in rate may suggest that active-species formation from **1** is dependent on the concentration of amine–borane, possibly aided by outer-sphere B–H⋅⋅⋅H–N interactions.[29] Overall, the current data suggest that if not the actual catalyst, dimeric species such as **5** likely sit close to the real catalyst.

**Scheme 6:**
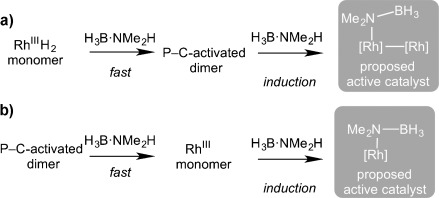
Proposed pathways for the formation of the active catalyst under conditions of high relative [H_3_B⋅NMe_2_H] (500 equiv) starting from a) monomeric and b) dimeric precatalysts.

In summary, P–C-activated dimeric complexes based upon the {Rh_2_(xantphos′)_2_}^2+^ motif are very active catalysts for the dehydrocoupling of H_3_B⋅NMe_2_H and the dehydropolymerization of H_3_B⋅NMeH_2_. Kinetic data suggest that the mechanisms of dehydrocoupling by dimeric and previously reported monomeric precatalysts may be closely related. The implication that dimeric species are active suggests that bimetallic cooperativity might be important for dehydropolymerization and offers opportunities to further tune catalyst properties as has successfully been demonstrated for olefin polymerization processes.[8] More generally given the wide use of xantphos as a ligand for many catalytic applications,[30] it will be interesting to see whether P–C-activated dimers prove to be a common motif in organometallic chemistry.

## Supporting Information

As a service to our authors and readers, this journal provides supporting information supplied by the authors. Such materials are peer reviewed and may be re-organized for online delivery, but are not copy-edited or typeset. Technical support issues arising from supporting information (other than missing files) should be addressed to the authors.

miscellaneous_information
