# Supplementary material for: P–C-Activated Bimetallic Rhodium Xantphos Complexes: Formation and Catalytic Dehydrocoupling of Amine–Boranes
Source: Angew Chem Int Ed Engl. 2015 Jul 3;54(35):10173–7. doi: 10.1002/anie.201504073 (PMC4557049; doi:10.1002/anie.201504073)
Supplement: Supplementary file 1 — miscellaneous_information [file anie0054-10173-sd1.pdf]

Angewandte Chemie
Eine Zeitschrift der Gesellschaft Deutscher Chemiker

Supporting Information

P–C-Activated Bimetallic Rhodium Xantphos Complexes: Formation and Catalytic Dehydrocoupling of Amine–Boranes**

Heather C. Johnson and Andrew S. Weller**

anie_201504073_sm_miscellaneous_information.pdf

Experimental S-2

Synthesis of new complexes S-3

Reaction between complex 5 and $H_3B{\cdot}NMe_2H$ (4 eq.) S-11

Addition of MeCN to complex 4 S-11

General procedures for amine-borane dehydrocoupling catalysis S-12

Kinetic plots S-13

Dehydropolymerisation of $H_3B{\cdot}NMeH_2$ S-21

X-ray crystallography S-21

References S-27

Experimental

All manipulations, unless otherwise stated, were performed under an argon atmosphere using standard Schlenk and glove-box techniques. Glassware was oven dried at 130 °C overnight and flamed under vacuum prior to use. Pentane, CH_2Cl_2 and MeCN were dried using a Grubbs type solvent purification system (MBraun SPS-800) and degassed by successive freeze-pump-thaw cycles.[1] C_6H_5F, 1,2-$C_6H_4F_2$ (pretreated with alumina) and CD_2Cl_2 were dried over CaH_2, vacuum distilled and stored over 3 Å molecular sieves. $H_3B·NMe_3$ and $H_3B·NMe_2H$ were purchased from Aldrich and sublimed prior to use (5 x 10^{-2} Torr, 298 K). $H_3B·NMeH_2$ was formed by a modification of the literature method.[2] [Rh($\kappa^2_{P,P}$-Xantphos)(nbd)][BAr^F_4] (nbd = norbornadiene),[3] [Rh($\kappa^2_{P,P}$-Xantphos)(η^2-$H_2B(CH_2CH_2{}^tBu·NMe_3)$)][$BAr^F_4$] (**1**),[4] [Rh($\kappa^3_{P,O,P}$-Xantphos)($PCy_3$)][$BAr^F_4$],[5] [Rh($\kappa^3_{P,O,P}$-Xantphos)$(H)_2$(NCMe)][$BAr^F_4$],[3] $D_3B·NMe_2H$,[6] $H_3B·NMe_2D$[6] and Na[$H_3B·NMe_2·BH_3$][7] were prepared by literature methods. NMR spectra were recorded on a Bruker AVIII-500 spectrometer at room temperature, unless otherwise stated. 1,2-Bis(diphenylphosphino)ethane (dppe) was purchased from Aldrich. In 1,2-$C_6H_4F_2$, ^1H NMR spectra were pre-locked to a sample of C_6D_6 (25%) and 1,2-$C_6H_4F_2$ (75%) and referenced to the centre of the downfield solvent multiplet, δ = 7.07. ^{31}P and ^{11}B NMR spectra were referenced against 85% H_3PO_4 (external) and $BF_3·OEt_2$ (external) respectively. Chemical shifts (δ) are quoted in ppm and coupling constants (J) in Hz. ESI-MS were recorded on a Bruker MicrOTOF instrument interfaced with a glove-box.[8] GC-MS was performed on a Waters GCT ToF mass spectrometer. Microanalyses were performed by Elemental Microanalysis Ltd. Gel permeation chromatography (GPC) was performed on a Viscotek RImax chromatograph, equipped with an automatic sampler, a pump, an injector and inline degasser. Fractionation was achieved with two T5000 columns that were contained within an oven at 35 °C. THF containing 0.1% w/w [$^nBu_4N$]Br was used as the eluent at a flow rate of 1.0 mL min^{-1}. Samples were dissolved in the eluent (2 mg mL^{-1}, unless otherwise stated), stirred for 1 h at room temperature and filtered with a Ministart SRP 15 filter (polytetrafluoroethylene membrane of 0.45 µm pore size) before analysis. The calibration was conducted using a series of monodisperse polystyrene standards obtained from Aldrich.

Synthesis of new complexes

[Rh_2($\kappa^2_{P,P}$-Xantphos´)$_2$(μ-η^2:η^2-$H_3B·NMe_2·BH_3$)][BAr^F_4] (4)

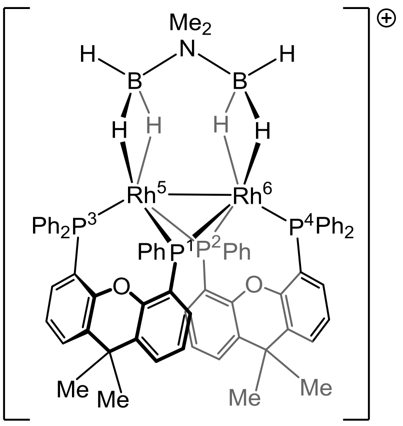

Figure S-1 Complex **4**. $[BAr^F_4]^-$ anion not shown.

In a Young's crystallisation flask containing **1** (20 mg, 0.012 mmol) dissolved in 0.5 mL 1,2-$C_6H_4F_2$, $H_3B·NMe_2H$ (13.8 mg, 0.234 mmol) dissolved in 0.6 mL 1,2-$C_6H_4F_2$ was added. Bubbling was observed immediately upon addition and the flask was sealed. After 12 hours, ^1H, $^{31}P\{^1H\}$ and ^{11}B NMR spectroscopies indicated that **4** was the major metal-containing product, and complete consumption of $H_3B·NMe_2H$ had occurred to yield $[H_2BNMe_2]_2$ as the major product of dehydrocoupling. The total volume of solution was reduced to ~ 0.5 mL *in vacuo* and pentane (5 mL) was added with stirring, resulting in a cloudy brown solution. On cooling to –78 °C, a brown solid precipitated. The yellow supernatant solution was decanted and the solid washed twice with pentane (2 x 3 mL), each time with sonication. The solid was recrystallised from 1,2-$C_6H_4F_2$ and pentane at 5 °C, from which orange crystals suitable for X-ray diffraction had grown, alongside brown oil. The oil showed NMR spectra suggestive of a mixture of **4** and decomposition products. Some crystals could be manually separated from the oil, yield: 5 mg (40%). These were nevertheless coated finely with oil and so were unsuitable for microanalysis. Similarly, oil-coated crystals of **4** can be formed in an analogous route using **5** (30 mg, *vide infra*) and 20 eq. $H_3B·NMe_2H$ (yield: 8 mg, 38%). Alternatively, addition of Na[$H_3B·NMe_2·BH_3$] to **5** forms **4** and Na[BAr^F_4] within 24 h, although **4** co-crystallised with Na[BAr^F_4] so material suitable for microanalysis was not obtained.

^{1}H NMR (500 MHz, CD_2Cl_2): δ 7.72 (br, 8H, $[BAr^F_4]^-$), 7.69 – 5.50 (m, 42H, Xantphos´ aryl groups), 7.55 (br, 4H, $[BAr^F_4]^-$), 4.14 (br, 2H, free HB), 2.47 (s, 6H, NMe_2), 1.79 (s, 6H, Xantphos´ CH_3), 1.24 (s,

6H, Xantphos´ CH_3), –2.68 (br, 2H, coordinated HB), –3.52 (br, 2H, coordinated HB). The signals at δ 4.14, –2.68 and –3.52 sharpen upon ^{11}B decoupling.

^{31}P{^{1}H} NMR (202 MHz, CD_2Cl_2): δ 108.5 (tt, phosphido groups), 13.3 (ddt, phosphino groups). Estimated coupling constants by gNMR[9] simulations: J_{13} = 10, J_{23} = 10, J_{14} = 10, J_{24} = 10, J_{51} = 112, J_{52} = 112, J_{53} = 122, J_{54} = 40, J_{61} = 112, J_{62} = 112, J_{63} = 40, J_{64} = 122, J_{56} = 4 (numbering is as in Figure S-1).

^{11}B NMR (160 MHz, CD_2Cl_2): δ 16.6 (br, BH_3), –6.6 (s, $[BAr^F_4]^-$).

ESI-MS (1,2-$C_6H_4F_2$, 60 ˚C, 4.5 kV): *m/z* [**4**]$^+$ 1280.23 (calc. 1280.24). Peak displays the expected isotopic pattern.

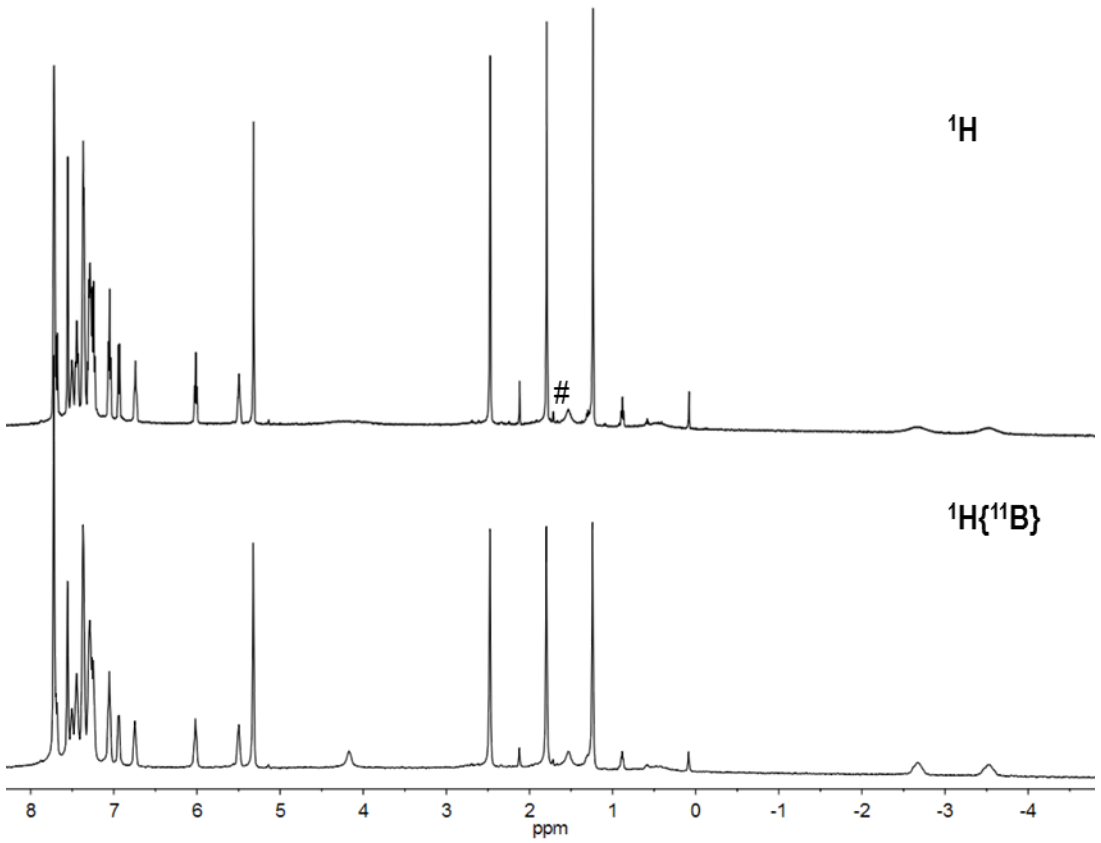

Figure S-2 ^{1}H (upper) and ^{1}H{^{11}B} (lower) NMR spectra of **4** in CD_2Cl_2. # = unidentified impurity.

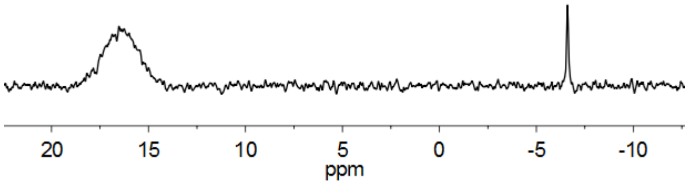

Figure S-3 Back-linear predicted ^{11}B NMR spectrum of **4** in CD_2Cl_2.

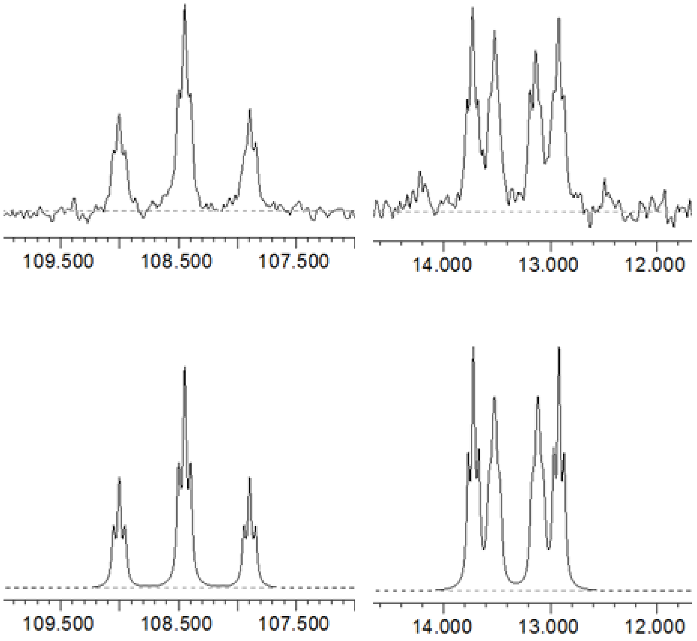

Figure S-4 Experimental (upper) and simulated (lower) $^{31}P\{^1H\}$ NMR spectra for **4** in CD_2Cl_2.

[Rh(κ^3$_{P,O,P}$-Xantphos´)(H$_3$B·NMe$_3$)]$_2$[BAr^F$_4$]$_2$ (5)

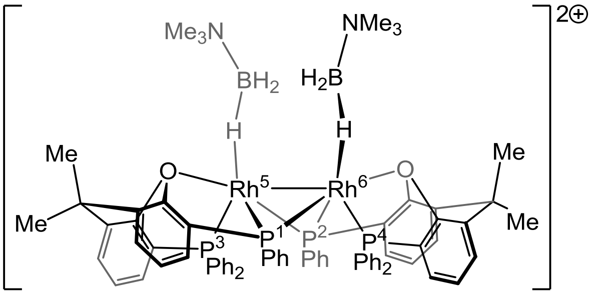

Figure S-5 Complex **5**. [BAr^F$_4$]$^-$ anions not shown.

To a Young's flask containing [Rh(κ^2$_{P,P}$-Xantphos)(nbd)][BAr^F$_4$] (100 mg, 0.061 mmol) and $H_3B·NMe_3$ (85 mg, 1.17 mmol), 1,2-$C_6H_4F_2$ was added (~ 3 mL). The flask was frozen in liquid N_2, the headspace evacuated and replaced with H_2 (*ca.* 4 atm) to form [Rh(κ^3$_{P,O,P}$-Xantphos)(H)$_2$(η^1-H$_3$B·NMe$_3$)][BAr^F$_4$] (**3**) on thawing and shaking. This mixture was degassed with three freeze-pump-thaw cycles, refilled with Ar, the flask sealed and heated to 40 °C. This initially formed a mixture of **2** and **3**, and periodic sampling of the reaction mixture for ^{31}P{^{1}H} NMR spectroscopy indicated complete conversion to **5** within 5 days, during which a dark red solution was formed. Alternatively, formation of **4** was complete within 48 h at 55 °C, although prolonged heating at this temperature caused decomposition to unidentified products. The volatiles were removed *in vacuo*, yielding dark red oil, which was washed and sonicated with pentane to form a dark red/orange solid. This was recrystallised from 1,2-$C_6H_4F_2$/pentane at 5 °C, affording crystals suitable for X-ray diffraction. Yield: 63 mg (67%).

^{1}H NMR (500 MHz, CD_2Cl_2): δ 7.75 – 6.53 (m, 42H, Xantphos´ aryl groups), 7.72 (br, 16H, [BAr^F$_4$]$^-$), 7.55 (br, 8H, [BAr^F$_4$]$^-$), 1.96 (s, 6H, Xantphos´ CH_3), 1.66 (s, 18H, NMe_3), 1.48 (s, 6H, Xantphos´ CH_3), –0.39 (br, 6H, H_3B). The signal at δ –0.39 sharpened upon ^{11}B decoupling.

^{31}P{^{1}H} NMR (202 MHz, CD_2Cl_2): δ 135.1 (tt, phosphido groups), 19.2 (ddt, phosphino groups). Estimated coupling constants by gNMR simulations: J_{13} = 10, J_{23} = 10, J_{14} = 10, J_{24} = 10, J_{51} = 125, J_{52} = 125, J_{53} = 112, J_{54} = 56, J_{61} = 125, J_{62} = 125, J_{63} = 56, J_{64} = 112, J_{56} = 4 (numbering is as shown in Figure S-5).

^{11}B NMR (160 MHz, CD_2Cl_2): δ –6.6 (s, [BAr^F$_4$]$^-$), –7.8 (br, BH_3).

ESI-MS was attempted but decomposition to unidentified species resulted.

Elemental Microanalysis: Calc. $Rh_2P_4O_2N_2B_4F_{48}C_{136}H_{102}$ (3081.19 g mol^{-1}): C, 53.02; H, 3.34; N, 0.91. Found: C, 52.91; H, 3.44; N, 1.00.

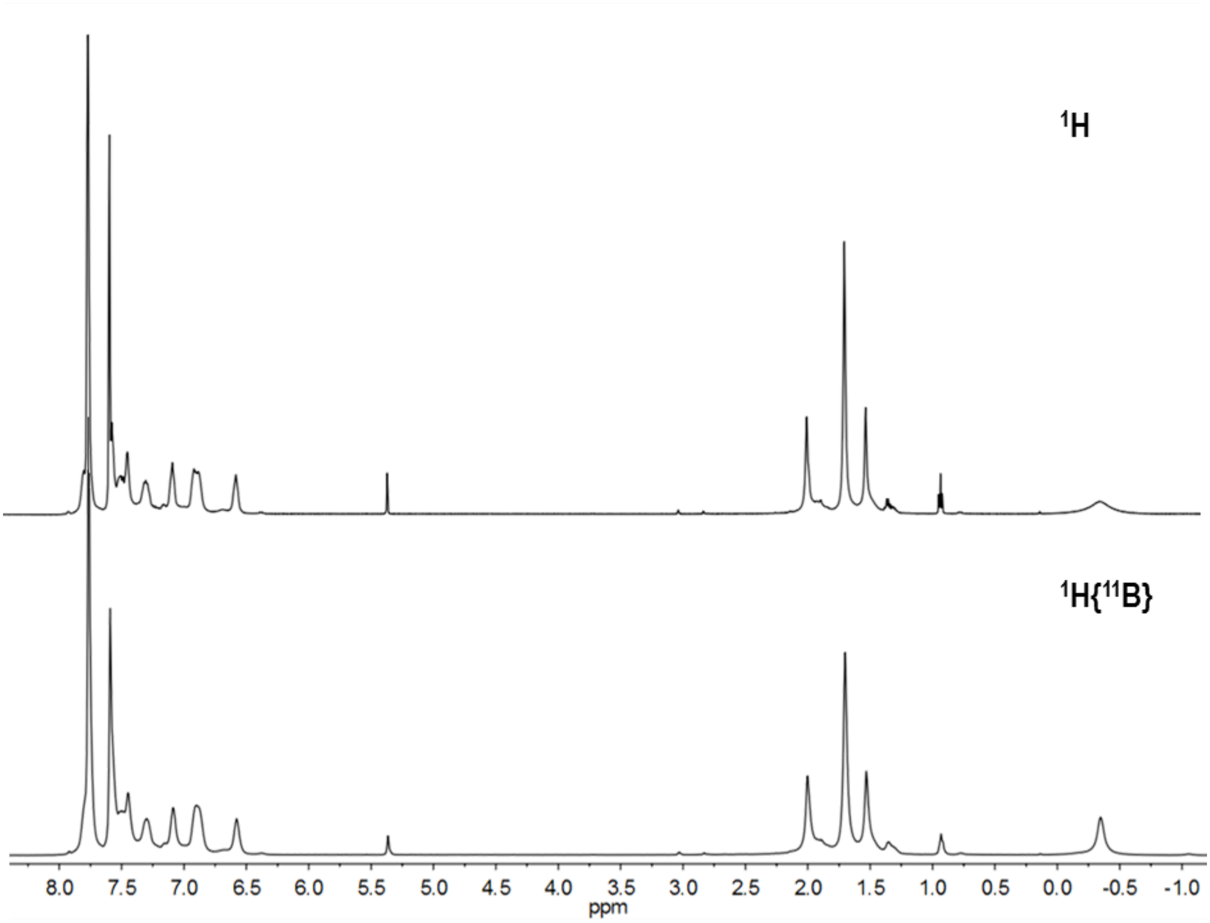

Figure S-6 ^{1}H (upper) and ^{1}H{^{11}B} (lower) NMR spectra of **5** in CD_2Cl_2.

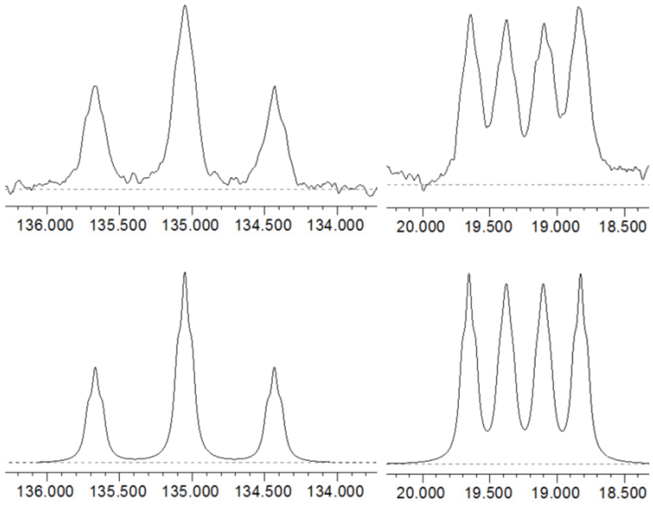

Figure S-7 Experimental (upper) and simulated (lower) ^{31}P{^{1}H} NMR spectrum of **5** in CD_2Cl_2.

[Rh(κ^3$_{P,O,P}$-Xantphos´)(NCMe)]$_2$[BAr^F$_4$]$_2$ (6)

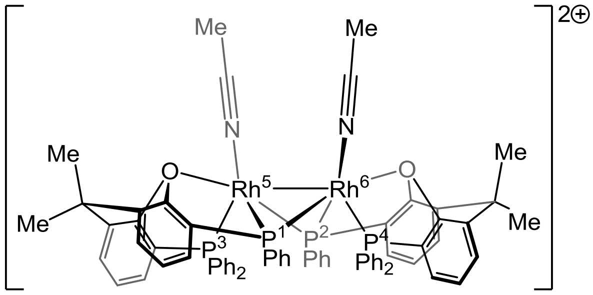

Figure S-8 Complex **6**. [BAr^F$_4$]$^-$ anions not shown.

Addition of MeCN to **5** in 1,2-$C_6H_4F_2$ or CD_2Cl_2 formed **6** immediately *in situ* (NMR spectroscopy). Attempts to recrystallize **6** resulted in the formation of orange oil.

^{1}H NMR (500 MHz, CD_2Cl_2): δ 7.71 (br, 16H, [BAr^F$_4$]$^-$), 7.65 – 6.71 (m, 42H, Xantphos´ aryl groups), 7.54 (br, 8H, [BAr^F$_4$]$^-$), 1.93 (s, 6H, Xantphos´ CH_3), 1.45 (s, 6H, Xantphos´ CH_3), 1.06 (s, 6H, MeCN).
^{31}P{^{1}H} NMR (202 MHz, CD_2Cl_2): δ 130.1 (tt, phosphido groups), 20.7 (ddt, phosphino groups). Estimated coupling constants by gNMR simulations: J_{13} = 10, J_{23} = 10, J_{14} = 10, J_{24} = 10, J_{51} = 122, J_{52} = 122, J_{53} = 104, J_{54} = 53, J_{61} = 122, J_{62} = 122, J_{63} = 53, J_{64} = 104, J_{56} = 5 (numbering is as in Figure S-8).
ESI-MS (1,2-$C_6H_4F_2$, 60 ˚C, 4.5 kV): Molecular ions observed at *m/z* 604.06 ([Rh(Xantphos´)]$_2$$^{2+}$, calc. 604.06, major), 624.57 ([{Rh(Xantphos´)}$_2$(NCMe)]$^{2+}$, calc. 624.57, mid), 645.09 ([**6**]$^{2+}$, calc. 645.09, minor).

[Rh_2($\kappa^3_{P,O,P}$-Xantphos´)$_2$(dppe)][BAr^F_4]$_2$ (7)

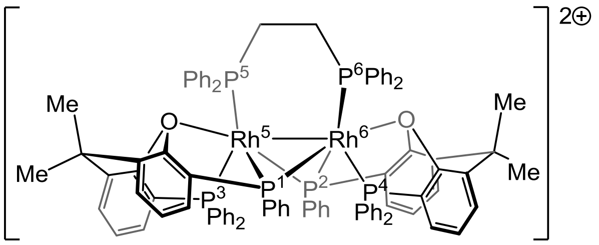

Figure S-9 Complex **7**. [BAr^F_4]$^-$ anions not shown.

To a mixture of **5** (15 mg, 0.005 mmol) and dppe (2 mg, 0.005 mmol), 1,2-$C_6H_4F_2$ was added, forming a red solution. Upon layering the solution with pentane at 5 °C, red crystals of **7** formed. Yield: 10 mg (62%).

^{1}H NMR (500 MHz, CD_2Cl_2): δ 7.79 – 5.83 (m, 62H, Xantphos´ and dppe aryl groups), 7.72 (br, 16H, [BAr^F_4]$^-$), 7.55 (br, 8H, [BAr^F_4]$^-$), 3.38 (m, 2H, 2 x CH, CH_2 chain), 2.19 (m, 2H, 2 x CH, CH_2 chain), 1.73 (s, 6H, Xantphos´ CH_3), 0.70 (s, 6H, Xantphos´ CH_3).

Upon ^{31}P decoupling, the signals at δ 3.38 and 2.19 collapsed to doublets ($^2J_{HH}$ = 12).

^{31}P{^{1}H} NMR (202 MHz, CD_2Cl_2): δ 134.1 (m, P1 and P2), 19.2 (m, P3 and P4), 3.9 (m, P5 and P6). Unfortunately, we were unable to simulate these signals satisfactorily. The peak at δ 134.1 was assigned on the basis of chemical shift as corresponding to bridging phosphido groups. A ^{1}H-^{31}P HMBC experiment showed a correlation between the signal at δ 3.9 and the dppe chain protons, assigning this signal as P5/P6.

ESI-MS (1,2-$C_6H_4F_2$, 60 ˚C, 4.5 kV): *m/z* [**7**]$^{2+}$ 803.63 (calc. 803.63). Peak displays the expected isotopic pattern.

Elemental Microanalysis: Calc. $Rh_2P_6O_2B_2F_{48}C_{156}H_{102}$ (3333.73 g mol^{-1}): C, 56.20; H, 3.08. Found: C, 56.07; H, 3.16.

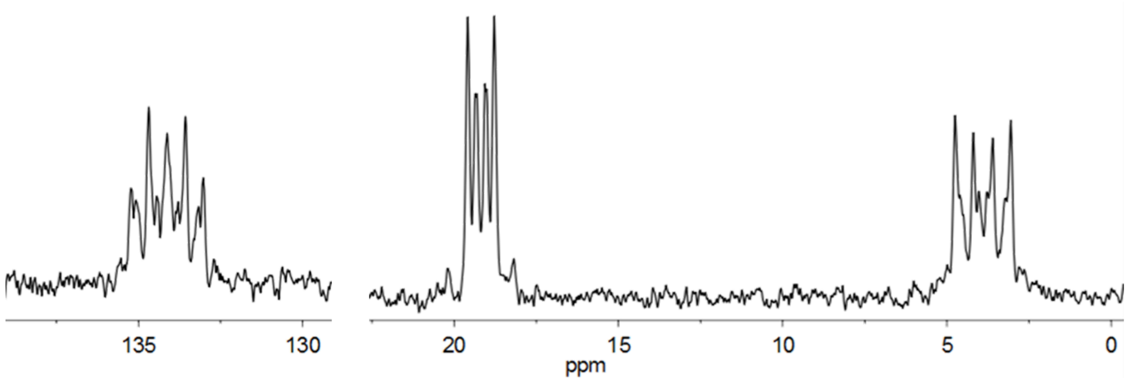

Figure S-10 $^{31}P\{^1H\}$ NMR spectrum of **7** (the range *ca.* δ 130 – 22 ppm has been omitted for clarity).

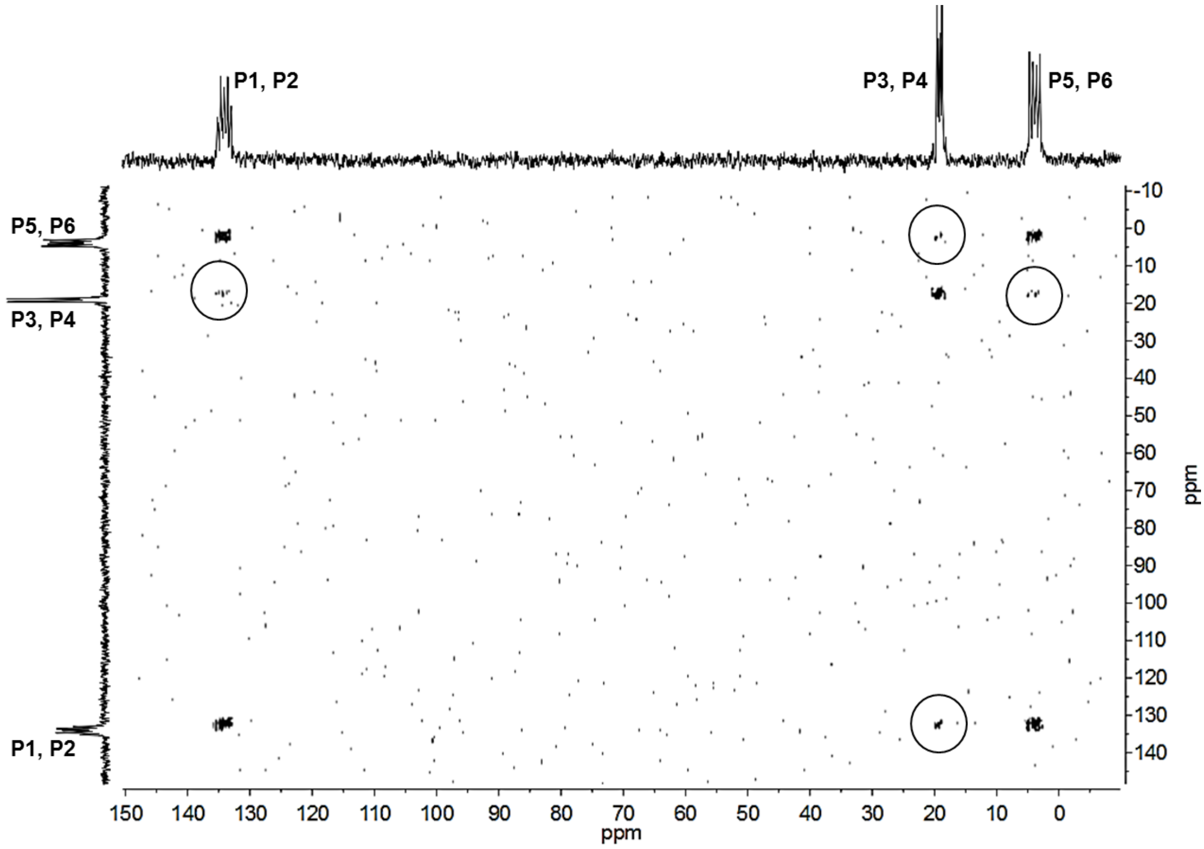

Figure S-11 ^{31}P-^{31}P COSY NMR spectrum of **7**. Circles are drawn to highlight the weaker cross peaks.

Reaction between complex 5 and $H_3B{\cdot}NMe_2H$ (4 eq.)

5 (10 mg, 0.003 mmol) and $H_3B{\cdot}NMe_2H$ (0.7 mg, 0.012 mmol) were each dissolved in 0.2 mL 1,2-$C_6H_4F_2$ and the two solutions were mixed in a high pressure NMR tube. The reaction was followed by ^1H, $^{31}P\{^1H\}$ and ^{11}B NMR spectroscopies. The major organometallic complex observed within 10 minutes was a complex consistent with the formulation $[Rh(Xantphos')(\eta^1\text{-}H_3B{\cdot}NMe_2H)]_2[BAr^F{}_4]_2$; in particular, two ^{31}P environments were observed in the $^{31}P\{^1H\}$ NMR spectrum at δ 135.0 and δ 18.6, and a broad signal in the ^1H NMR spectrum at δ –0.45 that sharpens upon ^{11}B decoupling was observed. These chemical shifts are very similar to those for **5** in 1,2-$C_6H_4F_2$ ($^{31}P\{^1H\}$ NMR: δ 135.3 and δ 19.1; ^1H NMR: δ –0.04), supporting this tentative assignment. After 2 hours (~ 30% consumption of $H_3B{\cdot}NMe_2H$), a mixture of this new complex and **5** were the major organometallic species observed by NMR spectroscopy. After 20 hours, the $H_3B{\cdot}NMe_2H$ had been fully consumed to form $[H_2BNMe_2]_2$, and a *ca.* 50:50 mixture of **4** and **5** had formed.

Addition of MeCN to complex 4

Excess MeCN (20 eq.) was added to *in situ* formed **4** (*via* dehydrocoupling of $H_3B{\cdot}NMe_2H$ by **5**) in a high pressure NMR tube. An intractable mixture of species was observed by ^1H and $^{31}P\{^1H\}$ NMR spectroscopies. No evidence for **6** was observed.

General procedures for amine-borane dehydrocoupling catalysis

Open system

In a typical experiment (*e.g.* 0.072 M $H_3B{\cdot}NMe_2H$, 0.1 mol% **5**), $H_3B{\cdot}NMe_2H$ (21.2 mg, 0.36 mmol) in 4.75 mL 1,2-$C_6H_4F_2$ was added to a 3-necked Schlenk flask with a magnetic stirrer bar. Under a flow of argon, an external mineral oil bubbler was connected and the argon flow adjusted to bubble at a rate of approximately 1.5 bubbles per second. In a separate flask, **5** (4.4 mg, 0.0014 mmol) was dissolved in 1 mL of 1,2-$C_6H_4F_2$. 0.25 mL of this precatalyst solution was injected *via* syringe into the 3-necked Schlenk flask. Catalysis was monitored by analysing regular aliquots of the reaction solution (0.1 mL samples, diluted with 0.25 mL 1,2-$C_6H_4F_2$ under argon, frozen in liquid N_2) by ^{11}B NMR spectroscopy. The reactions were performed at 298 K.

Closed system

To $H_3B{\cdot}NMe_2H$ (5.6 mg, 0.095 mmol), 1 mL of 1,2-$C_6H_4F_2$ was added. 0.3 mL was sampled and added to a high pressure NMR tube. To **5** (1.0 mg, 0.0003 mmol), 1 mL 1,2-$C_6H_4F_2$ was added. 0.1 mL was sampled and added to the high pressure NMR tube, resulting in a 0.072 M $H_3B{\cdot}NMe_2H$ solution with 0.1 mol% **5**. The reaction was followed *in situ* by ^{11}B NMR spectroscopy at 298 K.

Dehydropolymerisation of $H_3B{\cdot}NMeH_2$

To a Schlenk flask containing $H_3B{\cdot}NMeH_2$ (100 mg, 2.22 mmol) and a stirrer bar, 4.5 mL C_6H_5F was added. To a Young's NMR tube containing **5** (6.9 mg, 0.0022 mmol), 0.5 mL C_6H_5F was added, **5** was fully dissolved and transferred by cannula to the stirring solution of $H_3B{\cdot}NMeH_2$. The mixture was stirred under argon, open to a mercury bubbler, for the allotted time period, before quenching *via* syringe with 35 mL of hexanes. Following addition of hexanes, the $[H_2BNMeH]_n/H_3B{\cdot}NMeH_2$ mixture precipitated as an off-white solid at –78 ˚C. The yellow supernatant solution was filtered off, and the solid was dried for 2 minutes *in vacuo* to remove residual C_6H_5F. THF (2.5 mL) was added, dissolving the $[H_2BNMeH]_n/H_3B{\cdot}NMeH_2$ mixture, and this was filtered into a new Schlenk flask. Hexanes (40 mL) were added to the solution and cooled immediately to –78 ˚C, allowing the product to precipitate. The supernatant solution was removed by filtration, and the resulting solid was dried *in vacuo* for at least 12 hours before GPC analysis. Yield: 52 mg solid (containing ~15% unreacted $H_3BN{\cdot}MeH_2$, measured by ^{11}B NMR spectroscopy).

Kinetic plots

Effect of [$H_3B \cdot NMe_2H$] upon rate of 5-catalysed dehydrocoupling – open systems

Typical dehydrocoupling plots in open systems are shown in Figure S-12 ([$H_3B \cdot NMe_2H$]$_0$ = 0.288 M), Figure S-13 ([$H_3B \cdot NMe_2H$]$_0$ = 0.144 M), Figure S-14 ([$H_3B \cdot NMe_2H$]$_0$ = 0.072 M) and Figure S-15 ([$H_3B \cdot NMe_2H$]$_0$ = 0.018 M). The rates between the points in the graphs ranging from [$H_3B \cdot NMe_2H$]$_0$ = 0.288 M – 0.018 M (and duplicate runs, induction period excluded) were plotted *vs* [$H_3B \cdot NMe_2H$] to yield the saturation curve shown in Figure S-16. Addition of excess mercury to the reaction mixture after the induction period ([$H_3B \cdot NMe_2H$]$_0$ = 0.072 M) did not halt catalysis as has been observed in other systems,[10] suggesting homogeneous catalysis, although the total consumption of $H_3B \cdot NMe_2H$ was reduced to *ca.* 85%, and we suggest decomposition due to other factors.

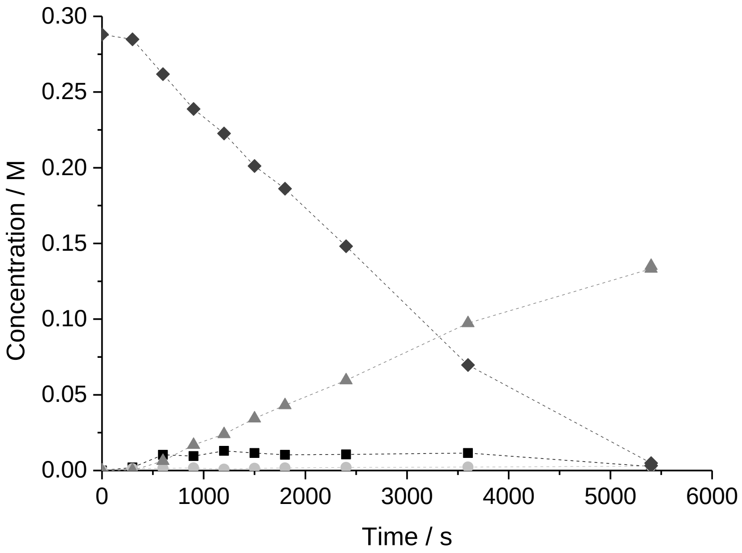

Figure S-12 Plot of concentration *vs* time (by ^{11}B NMR spectroscopy) of the dehydrocoupling of $H_3B \cdot NMe_2H$ by **5**. [$H_3B \cdot NMe_2H$]$_0$ = 0.288 M; [**5**] = 7.2 x 10^{-5} M; 1,2-$C_6H_4F_2$ solvent; open conditions. ◆ = $H_3B \cdot NMe_2H$; ■ = $H_2B{=}NMe_2$; ● = $HB(NMe_2)_2$; ▲ = [H_2BNMe_2]$_2$.

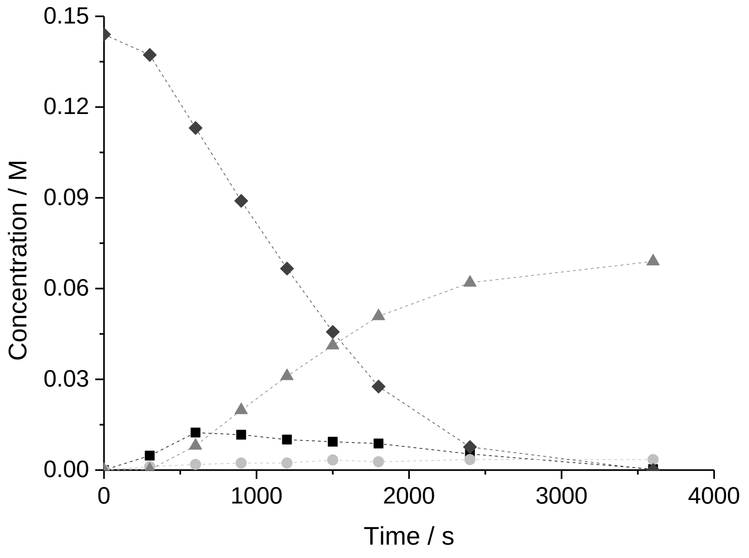

Figure S-13 Plot of concentration *vs* time (by ^{11}B NMR spectroscopy) of the dehydrocoupling of $H_3B{\cdot}NMe_2H$ by **5**. $[H_3B{\cdot}NMe_2H]_0$ = 0.144 M; [**5**] = 7.2 x 10^{-5} M; 1,2-$C_6H_4F_2$ solvent; open conditions. ◆ = $H_3B{\cdot}NMe_2H$; ■ = $H_2B{=}NMe_2$; ● = $HB(NMe_2)_2$; ▲ = $[H_2BNMe_2]_2$.

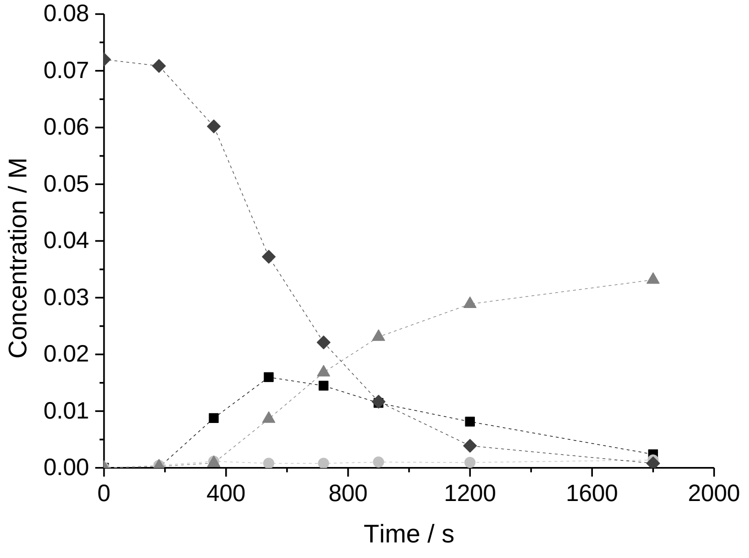

Figure S-14 Plot of concentration *vs* time (by ^{11}B NMR spectroscopy) of the dehydrocoupling of $H_3B{\cdot}NMe_2H$ by **5**. $[H_3B{\cdot}NMe_2H]_0$ = 0.072 M; [**5**] = 7.2 x 10^{-5} M; 1,2-$C_6H_4F_2$ solvent; open conditions. ◆ = $H_3B{\cdot}NMe_2H$; ■ = $H_2B{=}NMe_2$; ● = $HB(NMe_2)_2$; ▲ = $[H_2BNMe_2]_2$.

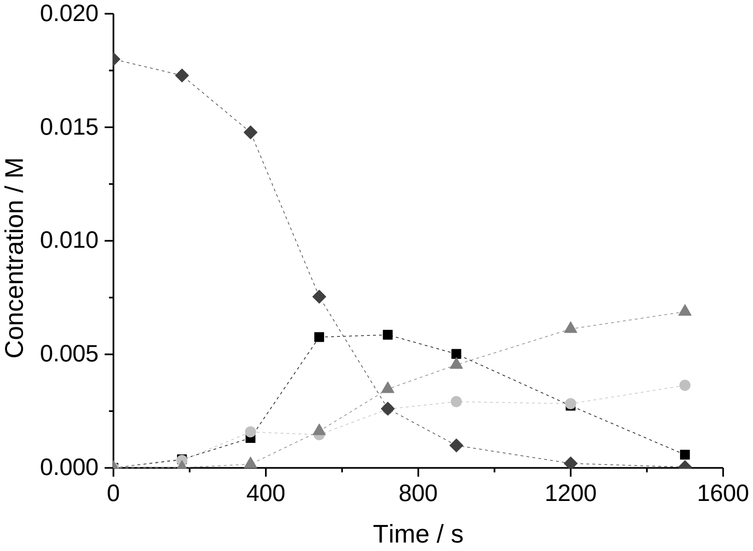

Figure S-15 Plot of concentration *vs* time (by ^{11}B NMR spectroscopy) of the dehydrocoupling of $H_3B{\cdot}NMe_2H$ by **5**. $[H_3B{\cdot}NMe_2H]_0$ = 0.018 M; [**5**] = 7.2 x 10^{-5} M; 1,2-$C_6H_4F_2$ solvent; open conditions. ◆ = $H_3B{\cdot}NMe_2H$; ■ = $H_2B{=}NMe_2$; ● = $HB(NMe_2)_2$; ▲ = $[H_2BNMe_2]_2$.

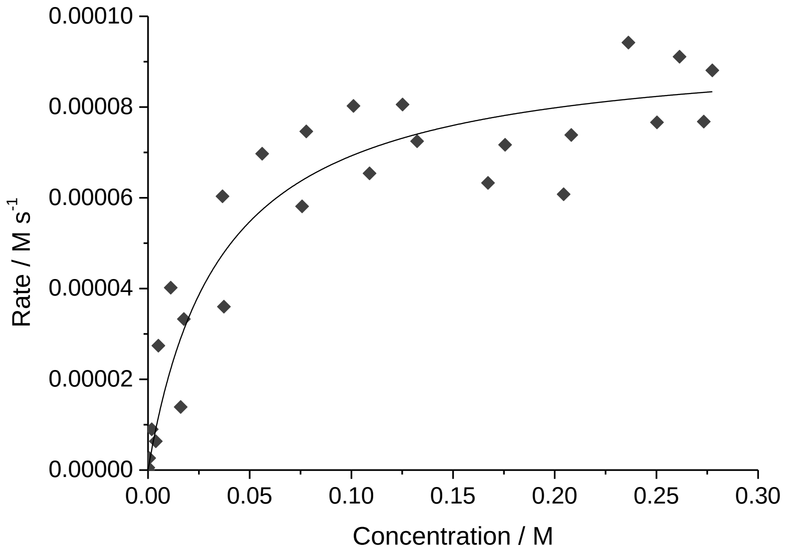

Figure S-16 Plot of [$H_3B{\cdot}NMe_2H$] *vs* rate of $H_3B{\cdot}NMe_2H$ consumption over the concentration range 0 to 0.27 M. Catalyst = **5**, 298 K, open system. Trendline is for illustration only. Rates measured by taking gradients between successive data points of ^{11}B concentration as measured by periodic sampling of the reaction. The scatter in the plot is a result of the measurements being taken over multiple separate runs.

Closed system

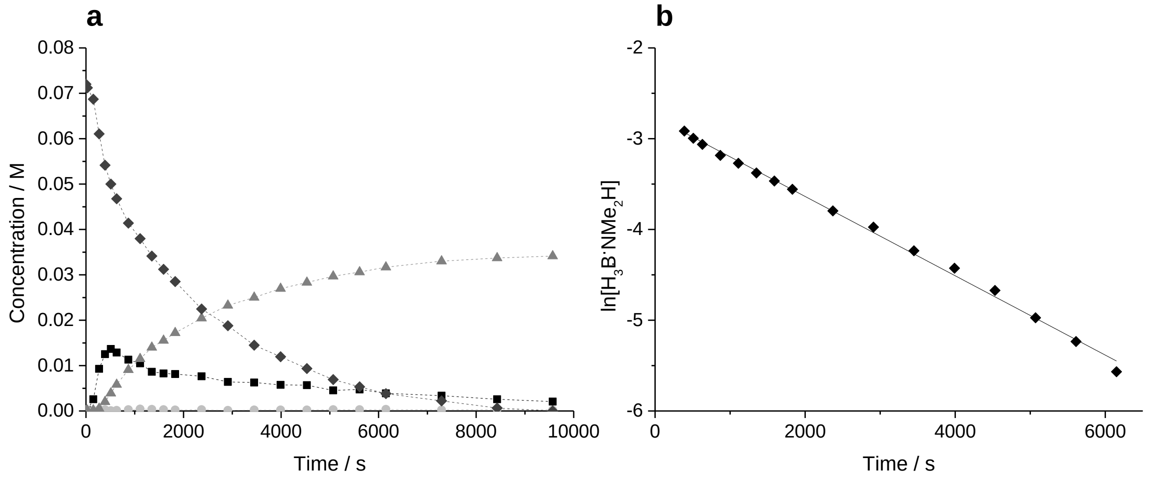

Figure S-17 (a) Plot of concentration *vs* time (by ^{11}B NMR spectroscopy) during the catalytic dehydrocoupling of $H_3B·NMe_2H$ (initial concentration 0.072 M) with [**5**] = 7.2 x 10^{-5} M. ◆ = $H_3B·NMe_2H$; ■ = $H_2B=NMe_2$; ● = $HB(NMe_2)_2$; ▲ = $[H_2BNMe_2]_2$. Sealed conditions. **(b)** Plot of ln[$H_3B·NMe_2H$] *vs* time during productive catalysis. Linear fit depicted by trendline. From trendline, k = (4.37 ± 0.07) x 10^{-4} s^{-1}. R^2 = 0.99656.

Kinetic isotope effects

Kinetic isotope effects were obtained from the zero order regions of the dehydrocoupling of $H_3B·NMe_2H$, $D_3B·NMe_2H$ and $H_3B·NMe_2D$ (initial concentrations of 0.288 M, [**5**] = 7.2 x 10^{-5} M, open conditions). A representative plot is shown in Figure S-18. Calculated kinetic isotope effects were 1.1 ± 0.2 for B—H/D substitution, and 2.0 ± 0.3 for N—H/D substitution.

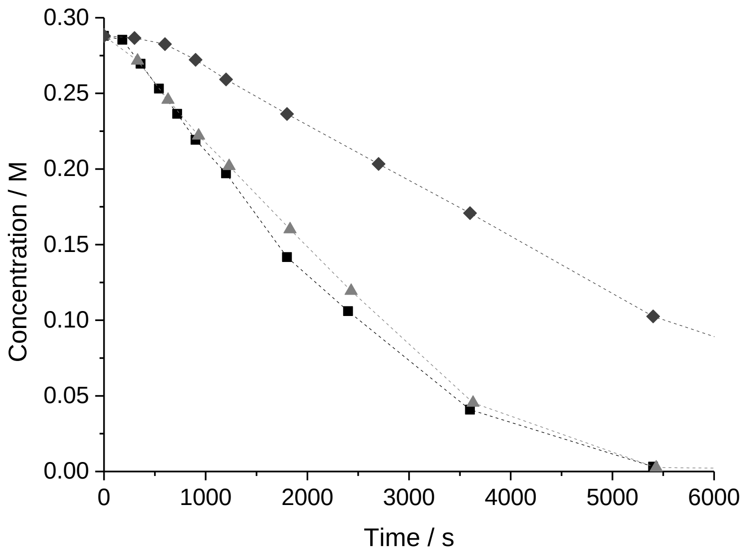

Figure S-18 Plot of concentrations *vs* time (by ^{11}B NMR spectroscopy) during separate catalytic dehydrocoupling reactions of $H_3B·NMe_2H$ (■), $D_3B·NMe_2H$ (▲) and $H_3B·NMe_2D$ (◆), each with [**5**] = 7.2 x 10^{-5} M and initial [amine-borane] = 0.288 M. First 6000 s shown.

Order in [Rh]

Figure S-19 shows a first order relationship between catalyst concentration and the rate of dehydrocoupling (zero order region).

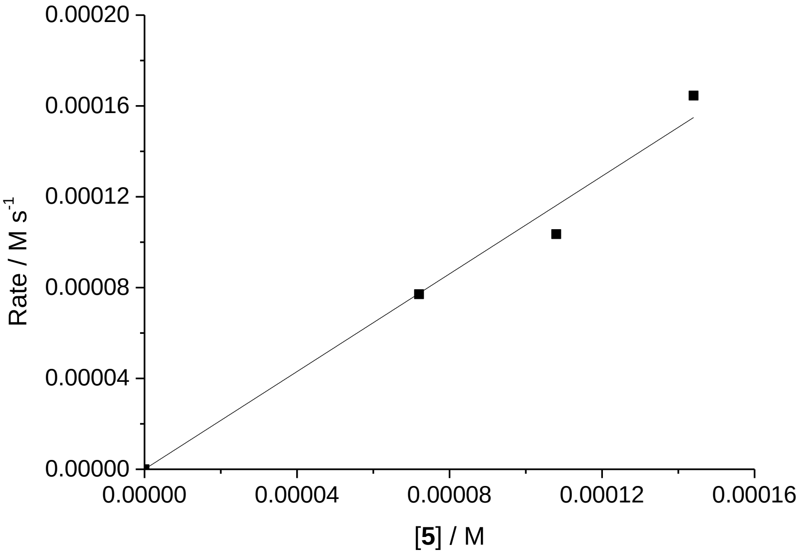

Figure S-19 Plot of rates *vs* [**5**] during separate catalytic dehydrocoupling reactions of $H_3B{\cdot}NMe_2H$ (initial concentration 0.144 M) upon altering the starting concentration of **5**. Rates obtained from the zero order region of the plots. Trendline shows line of best fit.

Dehydrocoupling catalysis using complex 4

Catalysis starting with complex **4** (Figure S-1) showed a longer induction period and a slower turnover frequency than under analogous conditions with complex **5** (Figure S-14), possibly due to the stronger coordination of $[H_3BNMe_2BH_3]^-$ *vs* H_3BNMe_3.

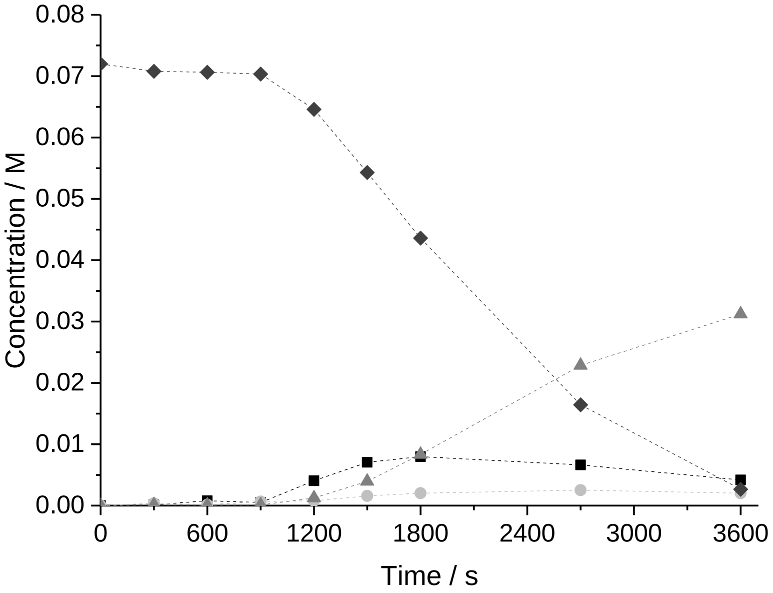

Figure S-20 Plot of concentration *vs* time (by ^{11}B NMR spectroscopy) of the dehydrocoupling of $H_3B{\cdot}NMe_2H$ by **4**. $[H_3B{\cdot}NMe_2H]_0$ = 0.072 M; [**4**] = 7.2 x 10^{-5} M; 1,2-$C_6H_4F_2$ solvent; open conditions. ◆ = $H_3B{\cdot}NMe_2H$; ■ = $H_2B{=}NMe_2$; ● = $HB(NMe_2)_2$; ▲ = $[H_2BNMe_2]_2$.

Dehydropolymerisation of $H_3B·NMeH_2$

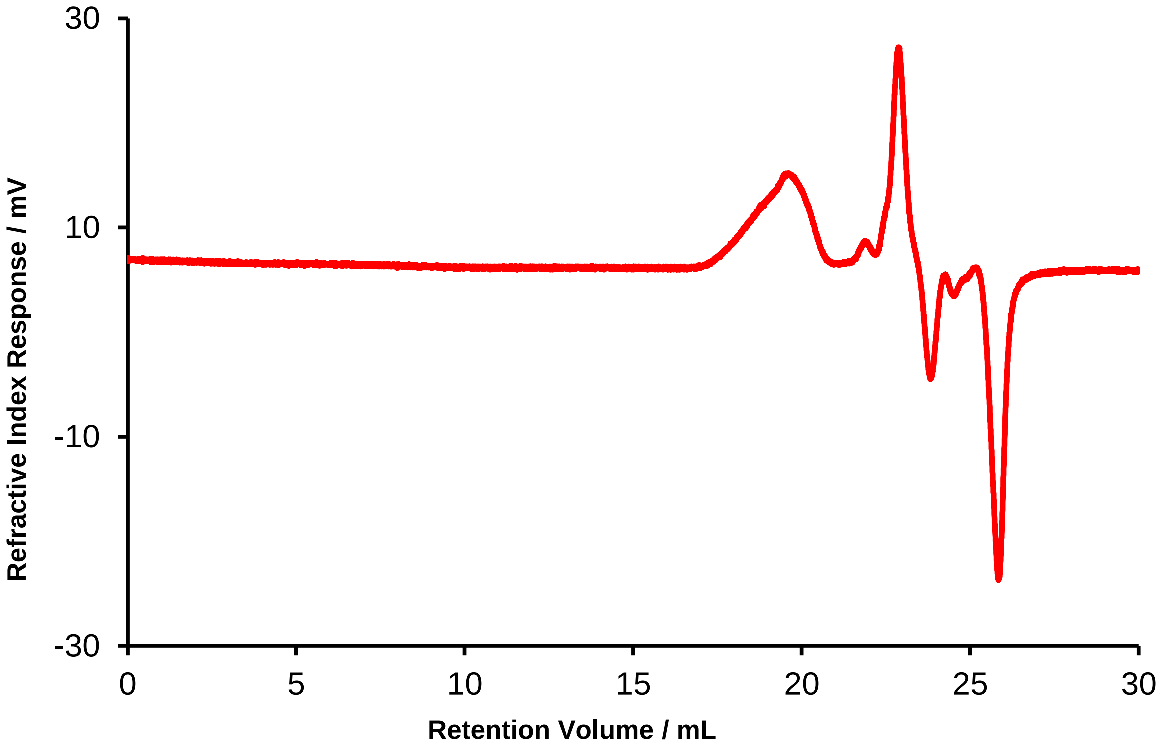

Figure S-21 Gel permeation chromatogram recorded for $[H_2BNMeH]_n$ at 2 mg/mL. By $^{11}B\{^1H\}$ NMR spectroscopy, 80% conversion to $[H_2BNMeH]_n$ was achieved after 2 hours of reaction with **5** (0.1 mol%), with the remainder being unreacted $H_3B·NMeH_2$. M_n = 28,700 g mol^{-1}; M_w = 47,500 g mol^{-1}.

X-ray crystallography

Relevant details about structure refinement are given in Table S-1. Data for **4**, **7** and **8** were collected on an Agilent Supernova diffractometer using graphite monochromated Cu Kα radiation (λ = 1.54180 Å) and a low temperature device; reduction and cell refinement was performed using CrysAlisPro.[11] Data for **5** were collected on a Enraf Nonious Kappa CCD difractometer using graphite monochromated Mo Kα radiation (λ = 0.71073 Å) and a low temperature device;[12] data were collected using COLLECT, reduction and cell refinement was performed using DENZO/SCALEPACK.[13] All structures were solved using Sir92[14] or Superflip.[15] All were refined using CRYSTALS.[16] Specific refinement details are given below.

Complex **4**

The $[BAr^F{}_4]^-$ anion exhibits considerable disorder, with two of the four aryl rings (and their corresponding CF_3 groups) each disordered over two sites. The rings were modelled over the two sites and their occupancies refined. In addition, a molecule of 1,2-$C_6H_4F_2$ solvent (0.3 occupancy) was located as part of the major component of one disordered ring, overlapping with the minor component. In addition, rotational disorder of one of the CF_3 groups in a non-disordered aryl ring was treated by modelling the fluorine atoms over two sites and restraining their geometry. Owing to the extensive disorder, some planarity and bond length restraints were used to give sensible structural parameters. A molecule of disordered pentane was also located, to which restraints were also applied.

All hydrogen atoms were located on the Fourier map, except those on the disordered pentane and 1,2-$C_6H_4F_2$. The hydrogen atoms on these molecules were placed in calculated positions. The hydrogen atoms were refined before RIDE restraints were added. The atoms H1/H2 and H4/H5 were placed riding upon B1 and B2, respectively.

Complex **5**

The Fourier difference map indicated the presence of diffuse electron density believed to be a molecule of the pentane solvent. SQUEEZE was used, leaving a void from which the electron density was removed. Rotational disorder of six of the CF_3 groups of the anion was treated by modelling the fluorine atoms over two sites and restraining their geometry. The hydrogen atoms were found on the Fourier map and refined before adding RIDE restraints. The atom H1 was placed riding upon B1.

Complex **7**

Rotational disorder of some of the CF_3 groups on the $[BAr^F{}_4]^-$ anions was treated by modelling the fluorine atoms over two sites and restraining their geometry. Hydrogen atoms were found on the Fourier map and refined before RIDE restraints were added.

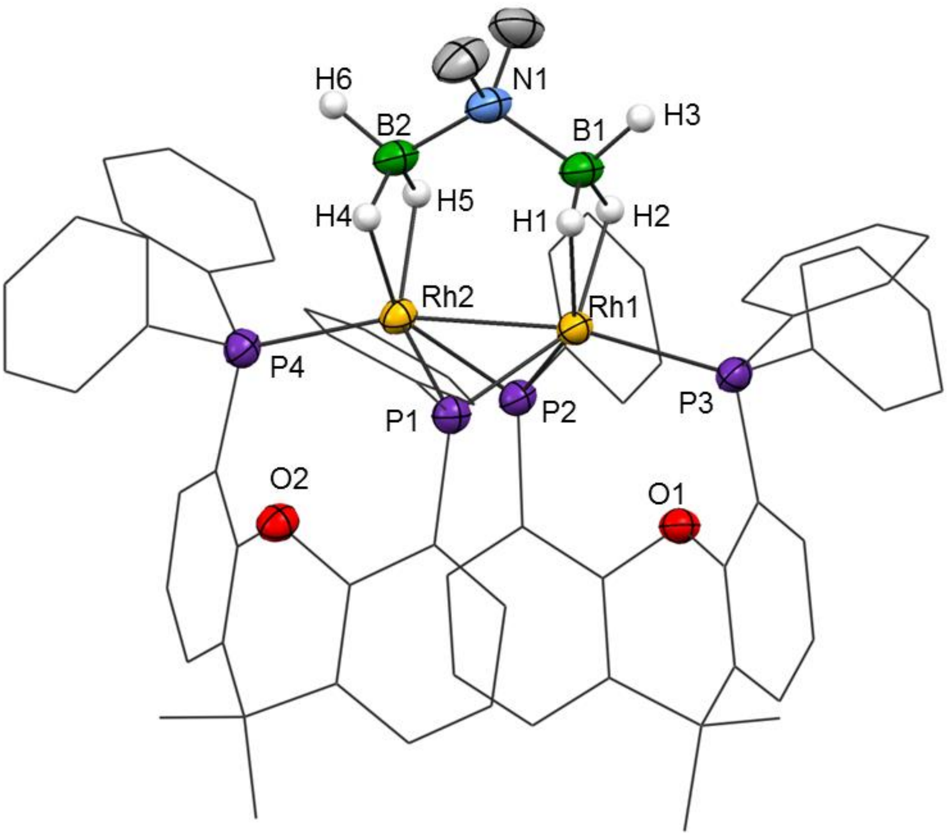

Figure S-22 Solid state structure of the cationic portion of **4**. Displacement ellipsoids are drawn at the 50% probability level. For clarity, carbon-bound H atoms are omitted, and the carbon atoms in the Xantphos´ ligands are depicted as a wireframe. Selected bond lengths (Å) and angles (°): Rh1-Rh2, 2.5928(4); Rh1-P1, 2.2455(12); Rh1-P2, 2.2500(11); Rh1-P3, 2.3427(11); Rh2-P1, 2.2461(11); Rh2-P2, 2.2663(11); Rh2-P4, 2.3325(11); Rh1-B1, 2.234(5); Rh2-B2, 2.229(5); Rh1-O1, 3.393(4); Rh2-O2, 3.412(4); B1-N1, 1.600(7); N1-B2, 1.583(7); P1-Rh2-P4, 114.64(4); P2-Rh1-P3, 110.89(4); B1-N1-B2, 117.5(4).

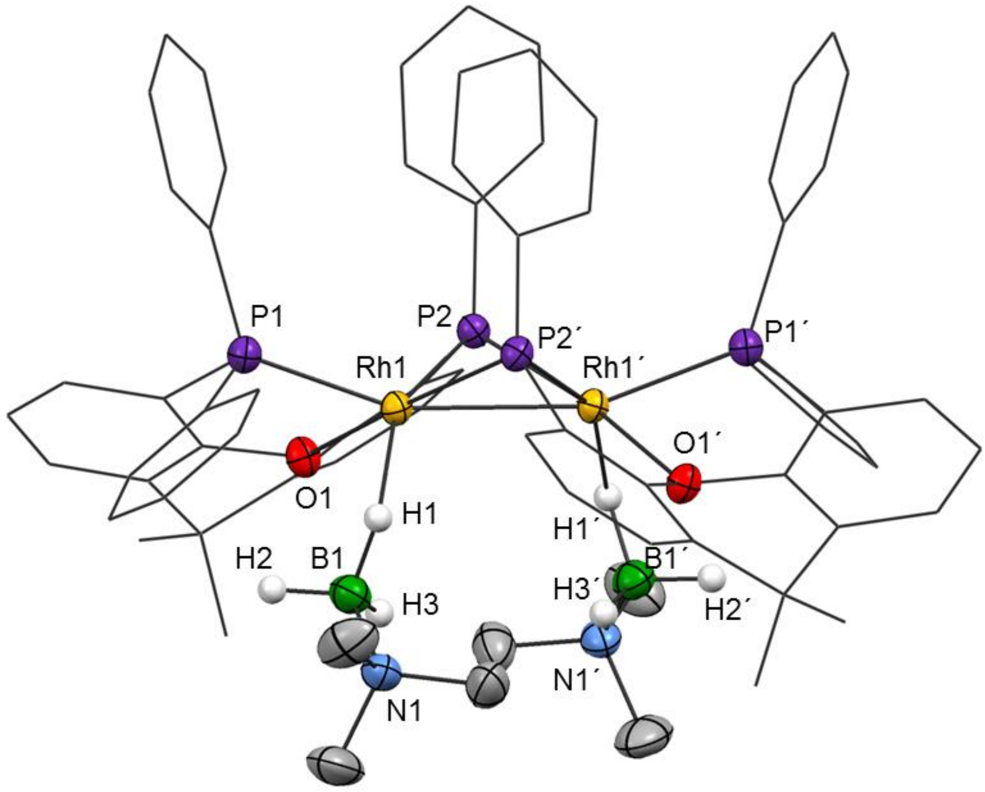

Figure S-23 Solid state structure of the cationic portion of **5**. Displacement ellipsoids are drawn at the 50% probability level. For clarity, H atoms are omitted, and the carbon atoms in Xantphos´ ligands are depicted as a wireframe. Selected bond lengths (Å) and angles (°): Rh1-Rh1´, 2.7965(5); Rh1-P2´, 2.1940(9); Rh1-P2, 2.2192(8); Rh1-P1, 2.3344(9); Rh1-O1, 2.288(2); Rh1-B1, 2.722(4); N1-B1, 1.594(5); P2-Rh1-P1, 119.72(3); Rh1-P2-Rh1´, 78.64(3); Rh1´-Rh1-P2´, 51.08(2); Rh1´-Rh1-P2, 50.28(2).

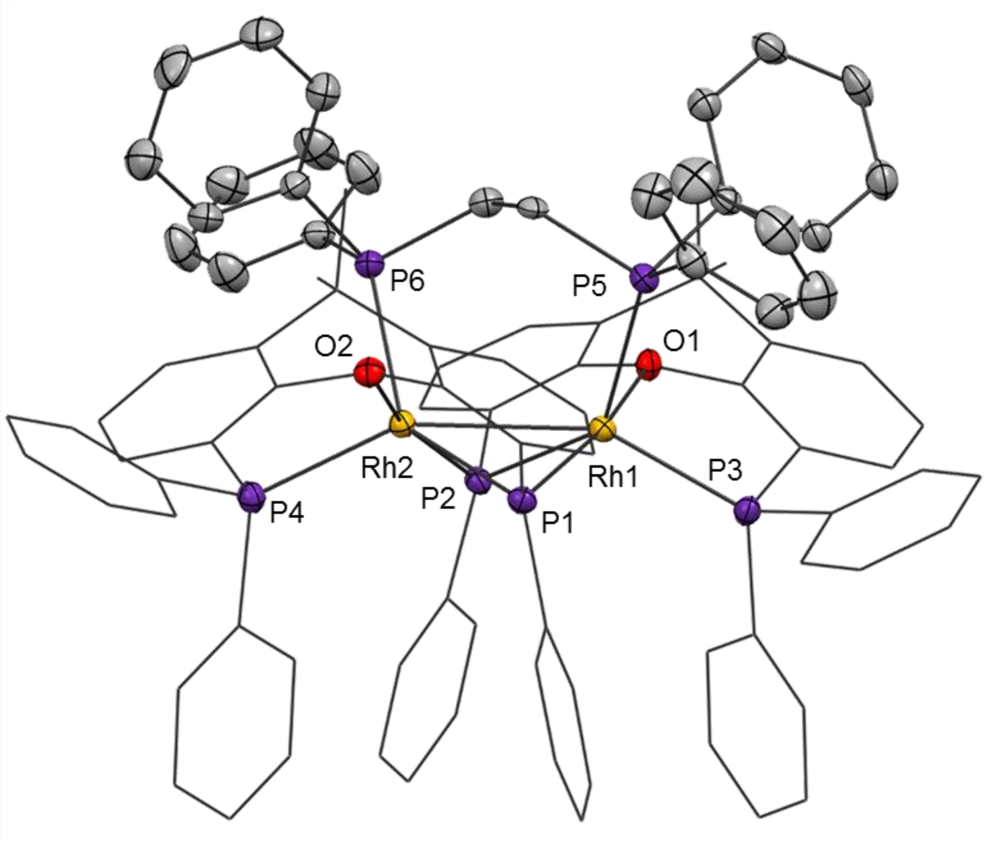

Figure S-24 Solid state structure of the cationic portion of **7**. Displacement ellipsoids are drawn at the 50% probability level. For clarity, H atoms are omitted, and the carbon atoms in Xantphos´ ligands are depicted as a wireframe. Selected bond lengths (Å) and angles (°): Rh1-Rh2, 2.8362(5); Rh1-P1, 2.2135(12); Rh1-P2, 2.2464(12); Rh1-P3, 2.3360(12); Rh1-P5, 2.4352(12); Rh1-O1, 2.309(3); Rh2-P1, 2.2571(12); Rh2-P2, 2.2194(12); Rh2-P4, 2.3565(12); Rh2-P6, 2.4165(12); Rh2-O2, 2.285(3); P2-Rh1-P3, 114.58(4); P1-Rh2-P4, 111.92(4); P6-Rh2-P4, 106.74(4); P6-Rh2-P1, 137.67(4); P6-Rh2-P2, 97.28(4); P5-Rh1-P3, 106.67(4); P5-Rh1-P1, 99.56(4); P5-Rh1-P2, 135.08(4).

Table S-1 Crystallographic data for **4**, **5**, and **7**.

	**4**	**5**	**7**
CCDC number	1062781	1062782	1062783
Formula	$C_{106.8}H_{91.2}B_3F_{24.6}NO_2P_4Rh_2$ $C_5H_{12}0.3(C_6H_4F_2)$	$C_{136}H_{102}B_4F_{48}N_2O_2P_4Rh_2$	$C_{156}H_{102}B_2F_{48}O_2P_6Rh_2$
M	2250.19	3081.17	3333.71
Crystal system	Triclinic	Monoclinic	Monoclinic
Space group	P -1	C 2/c	P 21/n
T [K]	150(2)	150(2)	150(2)
a [Å]	13.6309(2)	32.5926(3)	14.6815(2)
b [Å]	16.5228(3)	24.6249(3)	16.8673(2)
c [Å]	22.7975(4)	19.6743(2)	58.7574(5)
α [°]	94.0197(16)	90	90
β [°]	95.2079(14)	108.2170(5)	90.9227(8)
γ [°]	91.9282(14)	90	90
V [$Å^3$]	5096.56(15)	14999.0(3)	14548.6(3)
Z	2	4	4
Density [g cm^{-3}]	1.466	1.364	1.522
μ [mm^{-1}]	4.039	0.369	3.48
θ range [°]	3.200 ≤ θ ≤ 76.090	5.110 ≤ θ ≤ 27.489	3.115 ≤ θ ≤ 76.171
Reflns collected	61481	105008	167693
R_{int}	0.051	0.058	0.052
Completeness	98.80%	99.20%	99.30%
Data/restr/param	20971/1906/1622	17080/1140/1032	30115/1140/2085
R_1 [I > 2σ(I)]	0.0603	0.0644	0.0596
wR_2 [all data]	0.1695	0.171	0.1578
GoF	1.0108	0.9446	1.0967
Largest diff. pk and hole [e $Å^{-3}$]	2.41, -1.70	1.33, -1.13	1.17, -1.18

References

[1] A. B. Pangborn, M. A. Giardello, R. H. Grubbs, R. K. Rosen, F. J. Timmers, *Organometallics* **1996**, *15*, 1518-1520.

[2] N. Blaquiere, S. Diallo-Garcia, S. I. Gorelsky, D. A. Black, K. Fagnou, *J. Am. Chem. Soc.* **2008**, *130*, 14034-14035.

[3] R. J. Pawley, G. L. Moxham, R. Dallanegra, A. B. Chaplin, S. K. Brayshaw, A. S. Weller, M. C. Willis, *Organometallics* **2010**, *29*, 1717-1728.

[4] H. C. Johnson, C. L. McMullin, S. D. Pike, S. A. Macgregor, A. S. Weller, *Angew. Chem. Int. Ed.* **2013**, *52*, 9776-9780.

[5] H. C. Johnson, R. Torry-Harris, L. Ortega, R. Theron, J. S. McIndoe, A. S. Weller, *Catal. Sci. Tech.* **2014**, *4*, 3486-3494.

[6] M. E. Sloan, A. Staubitz, T. J. Clark, C. A. Russell, G. C. Lloyd-Jones, I. Manners, *J. Am. Chem. Soc.* **2010**, *132*, 3831-3841.

[7] H. Nöth, S. Thomas, *Eur. J. Inorg. Chem.* **1999**, 1373-1379.

[8] A. T. Lubben, J. S. McIndoe, A. S. Weller, *Organometallics* **2008**, *27*, 3303-3306.

[9] gNMR 5.1, Budzelaar, P. H. M., marketed by Adept Scientific until May 2005.

[10] a) C. A. Jaska, I. Manners, *J. Am. Chem. Soc.* **2004**, *126*, 9776-9785; b) C. A. Jaska, I. Manners, *J. Am. Chem. Soc.* **2004**, *126*, 1334-1335.

[11] *CrysAlisPro (Agilent Technologies, Oxford)* **2011**.

[12] J. Cosier, A. M. Glazer, *J. Appl. Crystallogr.* **1986**, *19*, 105-107.

[13] Z. Otwinowski, W. Minor, *Methods Enzymol.* **1997**, *276*, 307-326.

[14] A. Altomare, G. Cascarano, C. Giacovazzo, A. Guagliardi, M. C. Burla, G. Polidori, M. Camalli, *J. Appl. Crystallogr.* **1994**, *27*, 435.

[15] L. Palatinus, G. Chapuis, *J. Appl. Crystallogr.* **2007**, *40*, 786-790.

[16] P. W. Betteridge, J. R. Carruthers, R. I. Cooper, K. Prout, D. J. Watkin, *J. Appl. Crystallogr.* **2003**, *36*, 1487.
